# Supplementary material for: Ether glycerophospholipid control of peroxisome degradation confers protection from ischemia
Source: bioRxiv. 2026 Sep 29:2026.09.28.755133. Preprint. [Version 1] doi: 10.64898/2026.09.28.755133 (PMC13648633; doi:10.64898/2026.09.28.755133)
Supplement: Supplementary file 1 [file NIHPP2026.09.28.755133v1-supplement-1.pdf]

bioRxiv preprint doi: https://doi.org/10.64898/2026.09.28.755133; this version posted September 29, 2026. The copyright holder for this preprint (which was not certified by peer review) is the author/funder, who has granted bioRxiv a license to display the preprint in perpetuity. It is made available under a CC-BY 4.0 International license.

Supplementary Figure 1

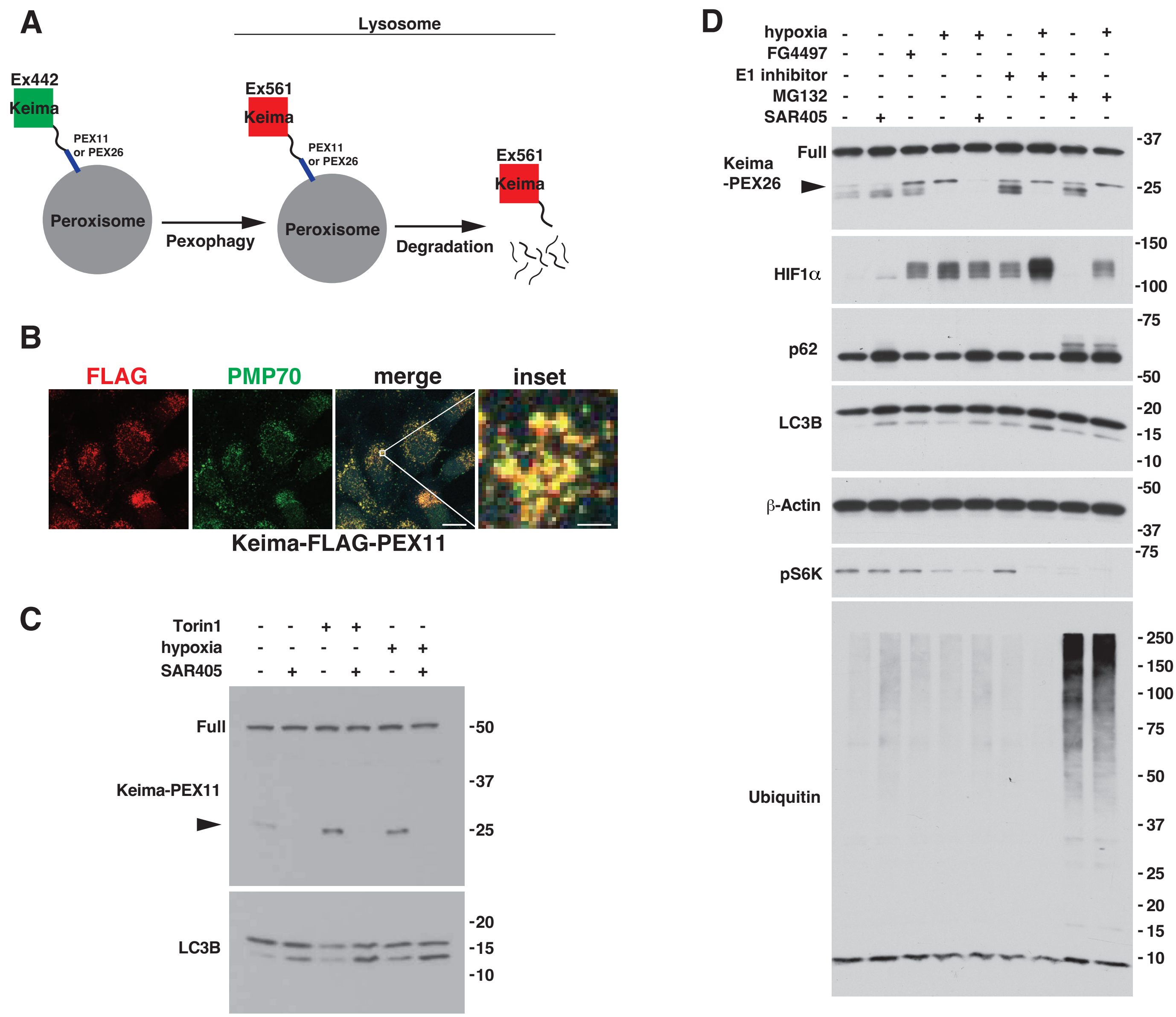

bioRxiv preprint doi: https://doi.org/10.64898/2026.09.28.755133; this version posted September 29, 2026. The copyright holder for this preprint (which was not certified by peer review) is the author/funder, who has granted bioRxiv a license to display the preprint in perpetuity. It is made available under a CC-BY 4.0 International license.

Supplementary Figure 2

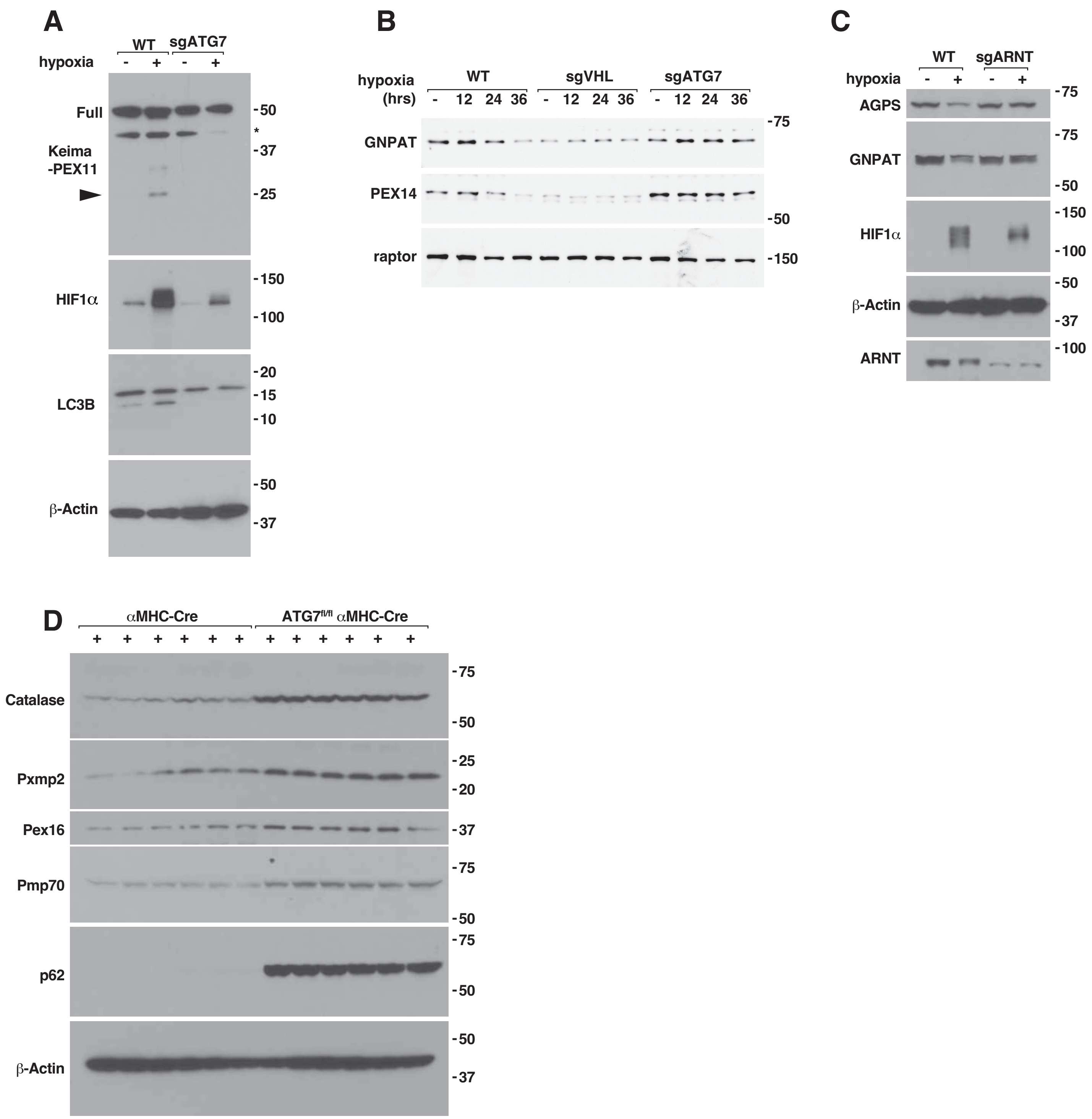

bioRxiv preprint doi: https://doi.org/10.64898/2026.09.28.755133; this version posted September 29, 2026. The copyright holder for this preprint (which was not certified by peer review) is the author/funder, who has granted bioRxiv a license to display the preprint in perpetuity. It is made available under a CC-BY 4.0 International license.

Supplementary Figure 3

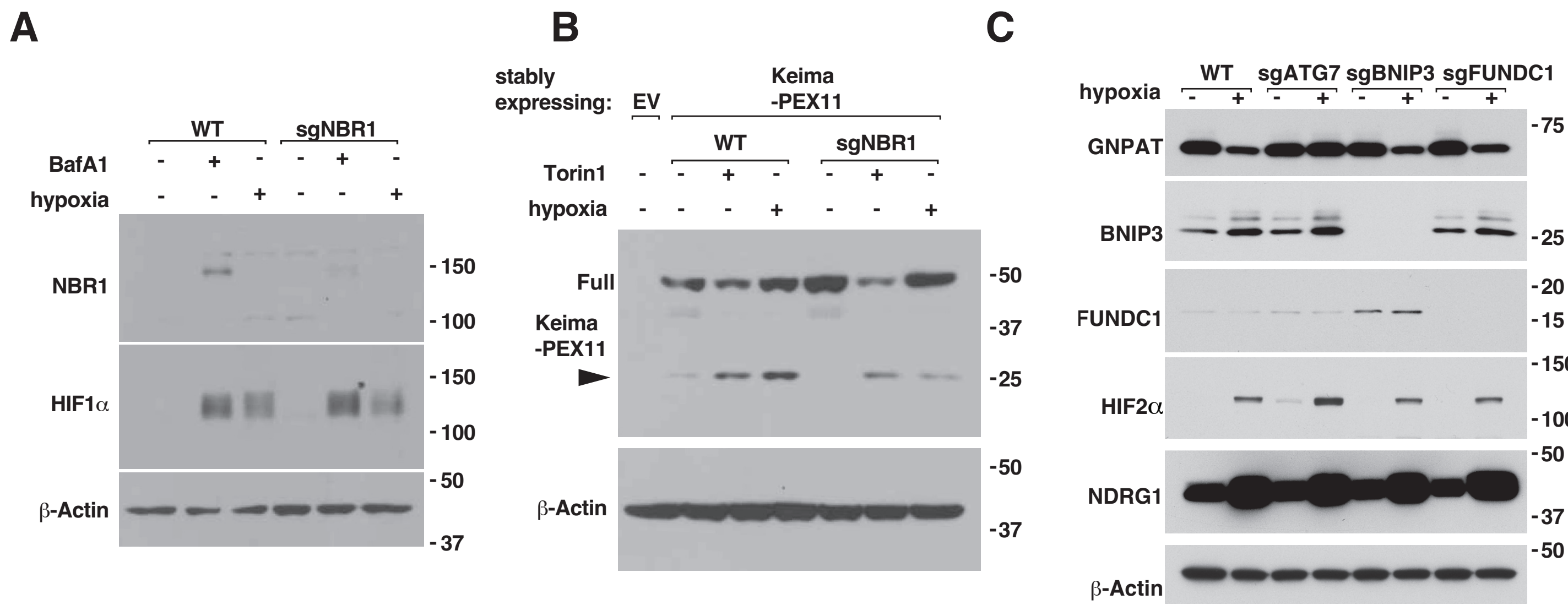

bioRxiv preprint doi: https://doi.org/10.64898/2026.09.28.755133; this version posted September 29, 2026. The copyright holder for this preprint (which was not certified by peer review) is the author/funder, who has granted bioRxiv a license to display the preprint in perpetuity. It is made available under a CC-BY 4.0 International license.

Supplementary Figure 4

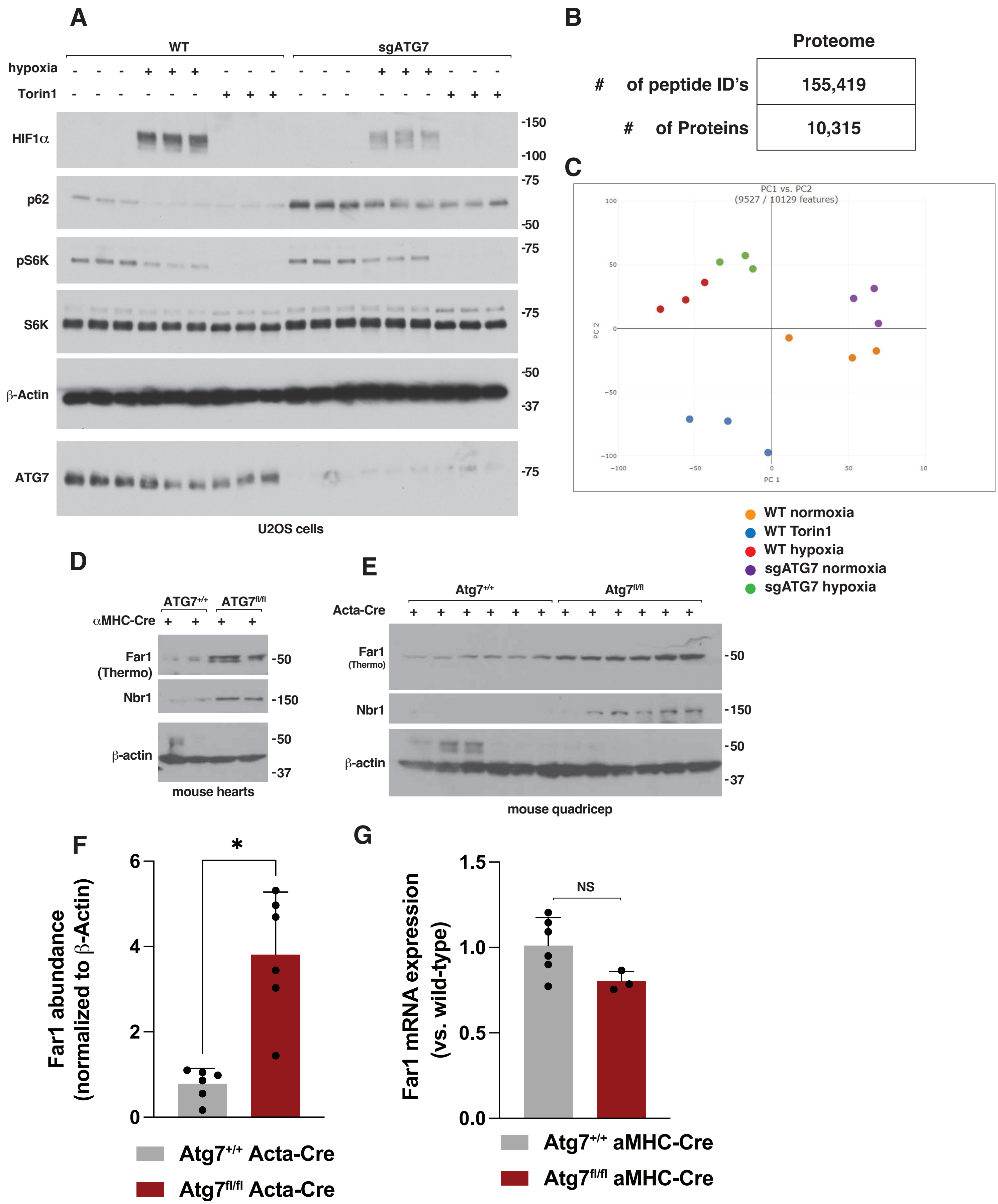

bioRxiv preprint doi: https://doi.org/10.64898/2026.09.28.755133; this version posted September 29, 2026. The copyright holder for this preprint (which was not certified by peer review) is the author/funder, who has granted bioRxiv a license to display the preprint in perpetuity. It is made available under a CC-BY 4.0 International license.

Supplementary Figure 5

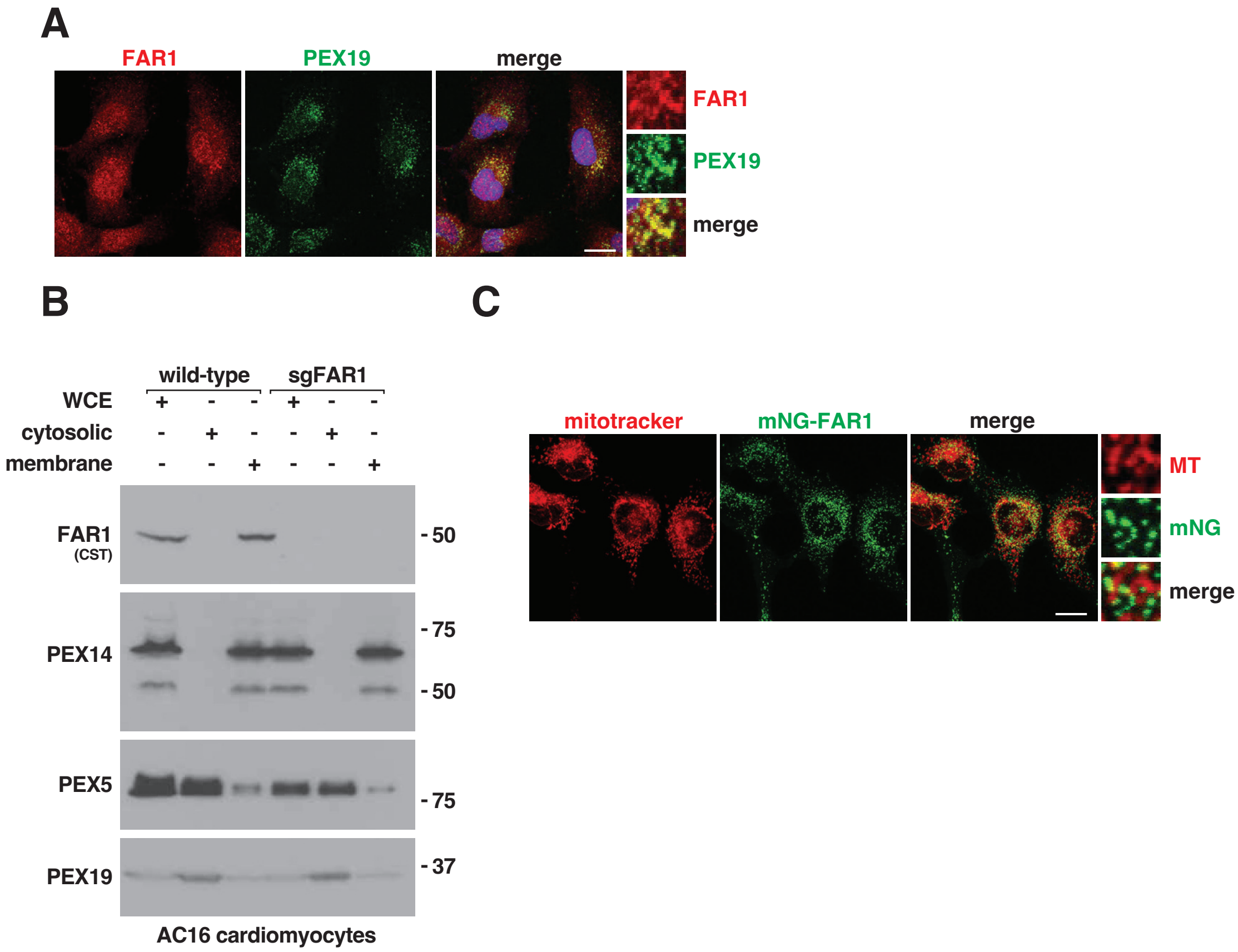

bioRxiv preprint doi: https://doi.org/10.64898/2026.09.28.755133; this version posted September 29, 2026. The copyright holder for this preprint (which was not certified by peer review) is the author/funder, who has granted bioRxiv a license to display the preprint in perpetuity. It is made available under a CC-BY 4.0 International license.

Supplementary Figure 6

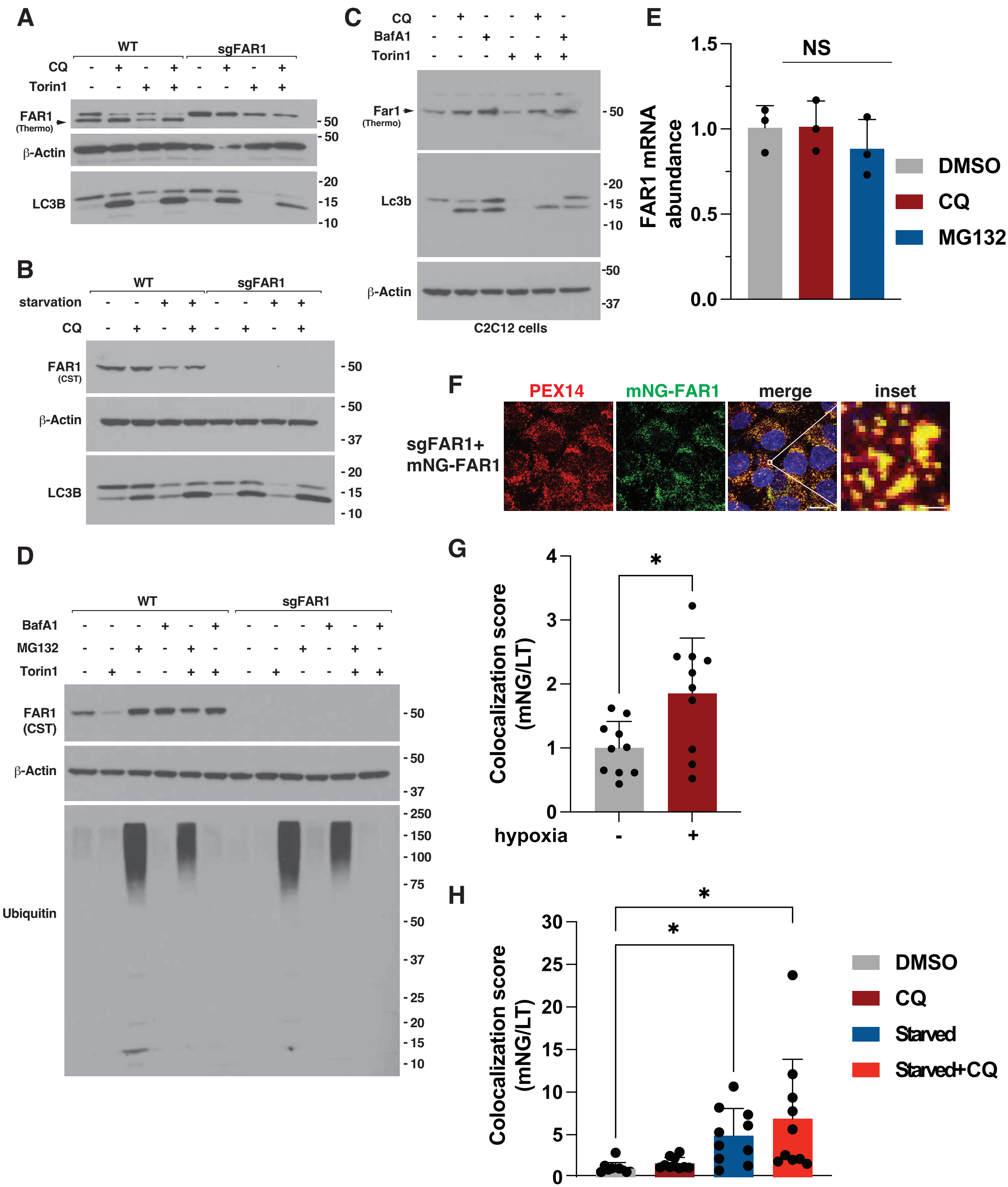

bioRxiv preprint doi: https://doi.org/10.64898/2026.09.28.755133; this version posted September 29, 2026. The copyright holder for this preprint (which was not certified by peer review) is the author/funder, who has granted bioRxiv a license to display the preprint in perpetuity. It is made available under a CC-BY 4.0 International license.

Supplementary Figure 7

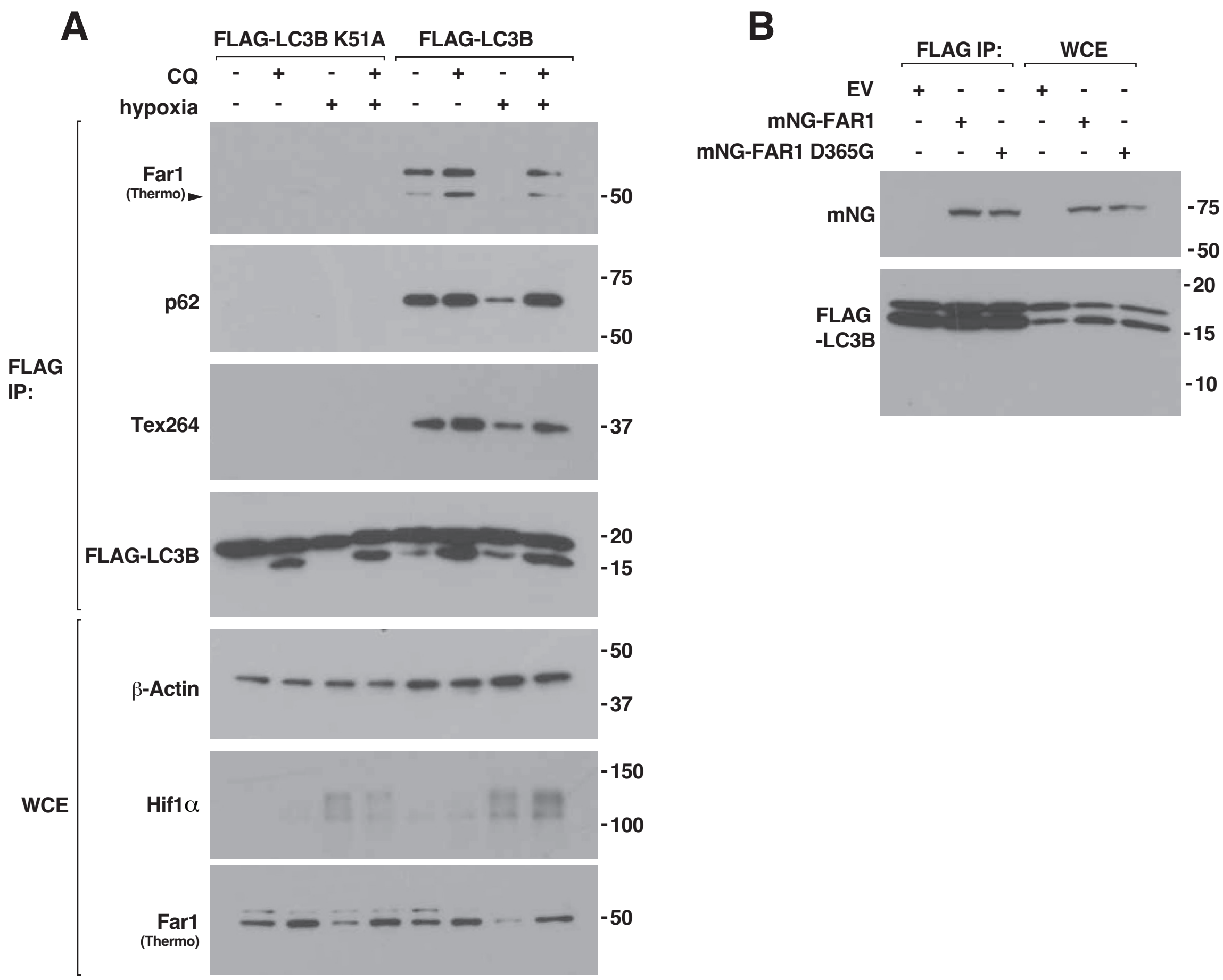

bioRxiv preprint doi: https://doi.org/10.64898/2026.09.28.755133; this version posted September 29, 2026. The copyright holder for this preprint (which was not certified by peer review) is the author/funder, who has granted bioRxiv a license to display the preprint in perpetuity. It is made available under a CC-BY 4.0 International license.

Supplementary Figure 8

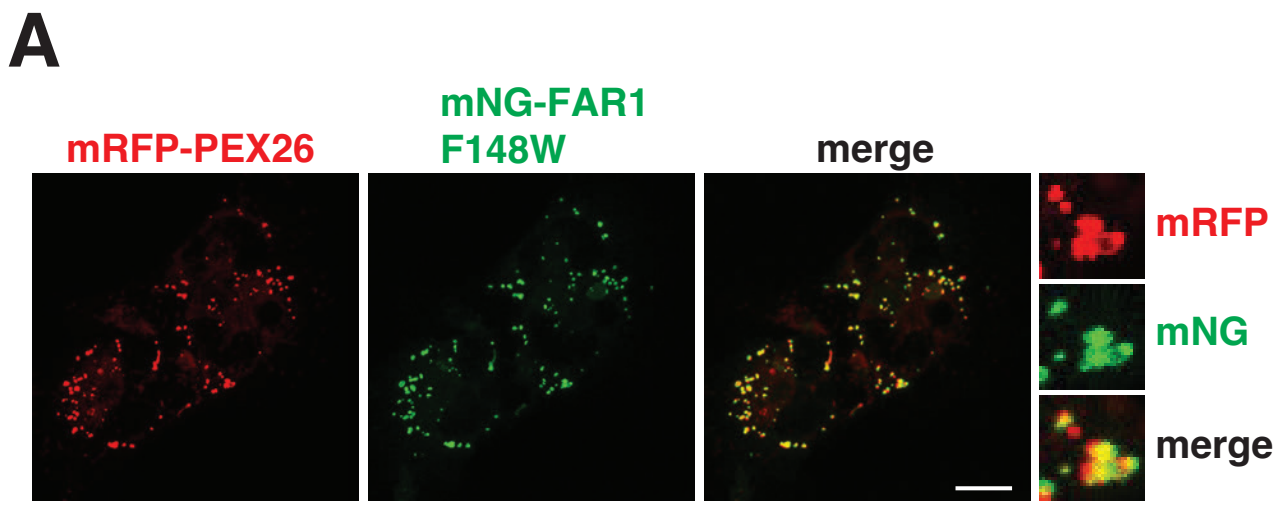

bioRxiv preprint doi: https://doi.org/10.64898/2026.09.28.755133; this version posted September 29, 2026. The copyright holder for this preprint (which was not certified by peer review) is the author/funder, who has granted bioRxiv a license to display the preprint in perpetuity. It is made available under a CC-BY 4.0 International license.

Supplementary Figure 9

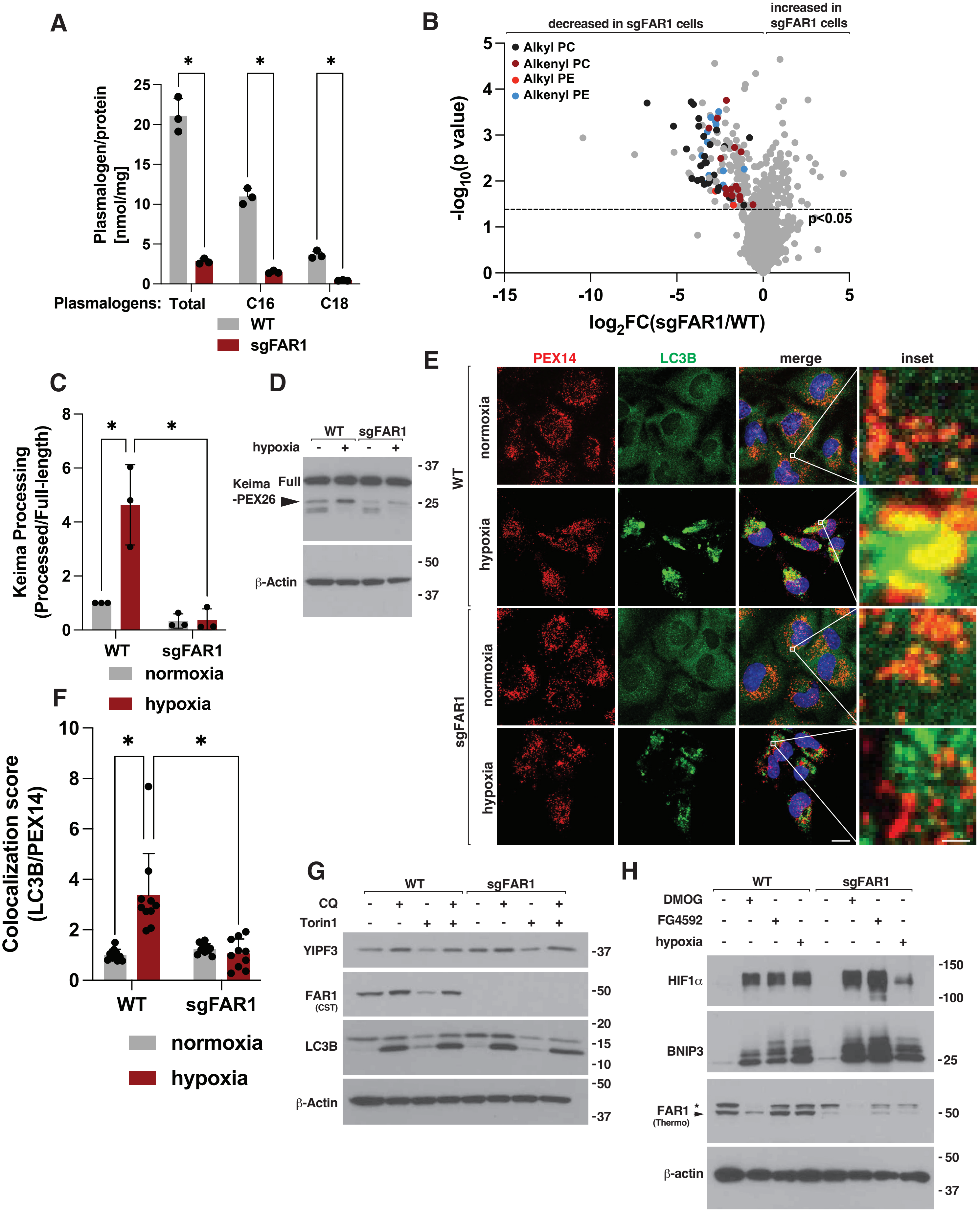

bioRxiv preprint doi: https://doi.org/10.64898/2026.09.28.755133; this version posted September 29, 2026. The copyright holder for this preprint (which was not certified by peer review) is the author/funder, who has granted bioRxiv a license to display the preprint in perpetuity. It is made available under a CC-BY 4.0 International license.

Supplementary Figure 10

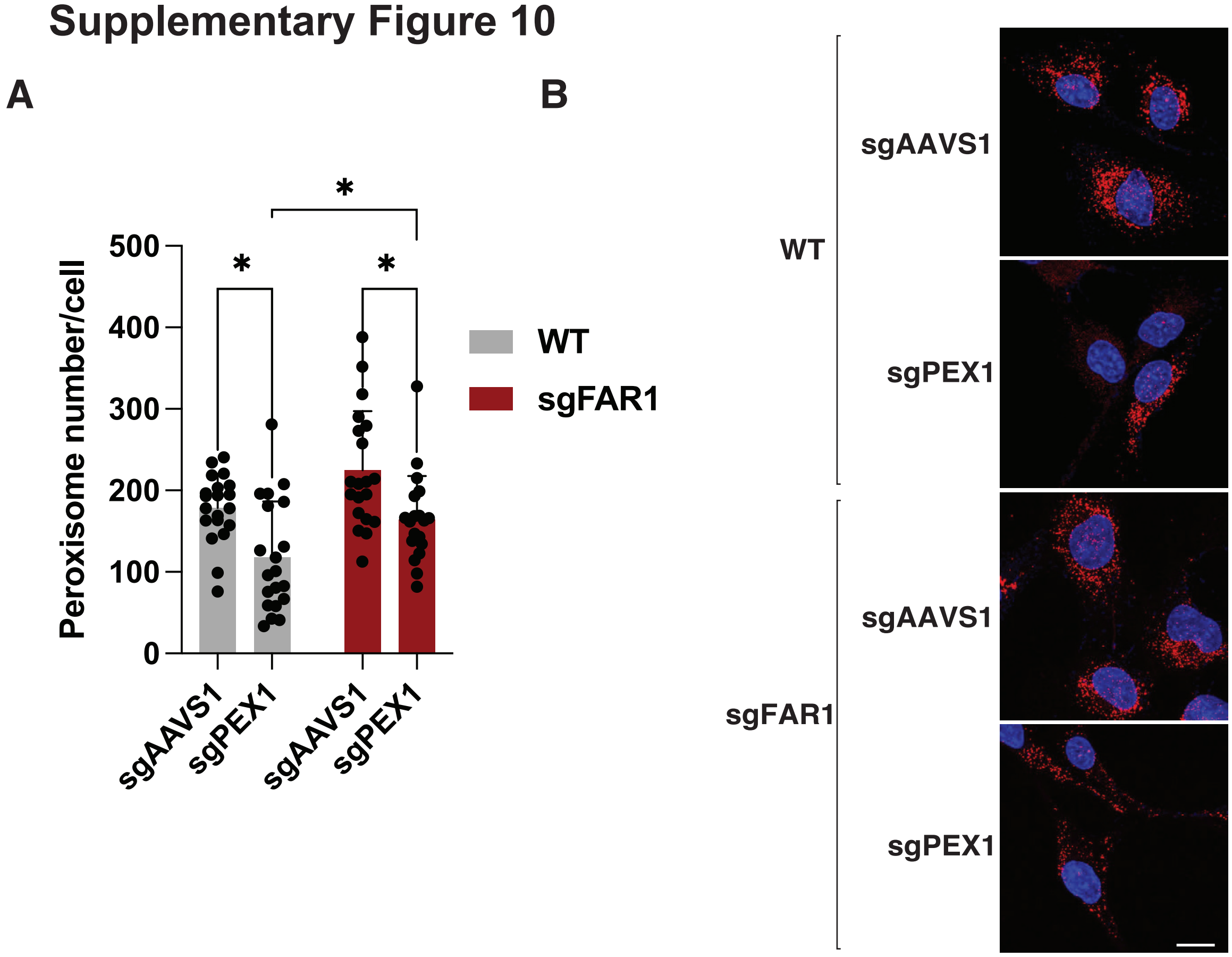

bioRxiv preprint doi: https://doi.org/10.64898/2026.09.28.755133; this version posted September 29, 2026. The copyright holder for this preprint (which was not certified by peer review) is the author/funder, who has granted bioRxiv a license to display the preprint in perpetuity. It is made available under a CC-BY 4.0 International license.

Supplementary Figure 11

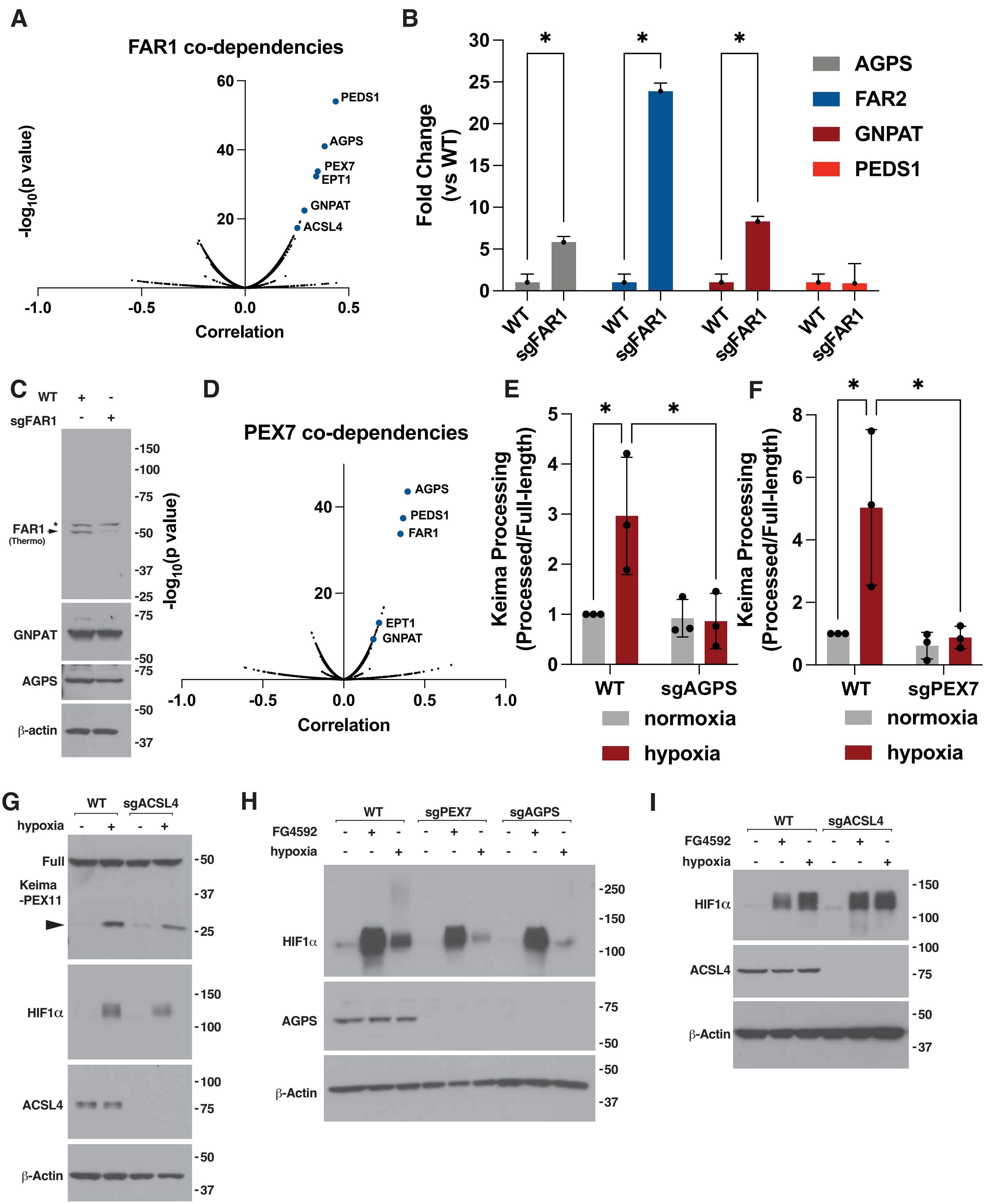

bioRxiv preprint doi: https://doi.org/10.64898/2026.09.28.755133; this version posted September 29, 2026. The copyright holder for this preprint (which was not certified by peer review) is the author/funder, who has granted bioRxiv a license to display the preprint in perpetuity. It is made available under a CC-BY 4.0 International license.

Supplementary Figure 12

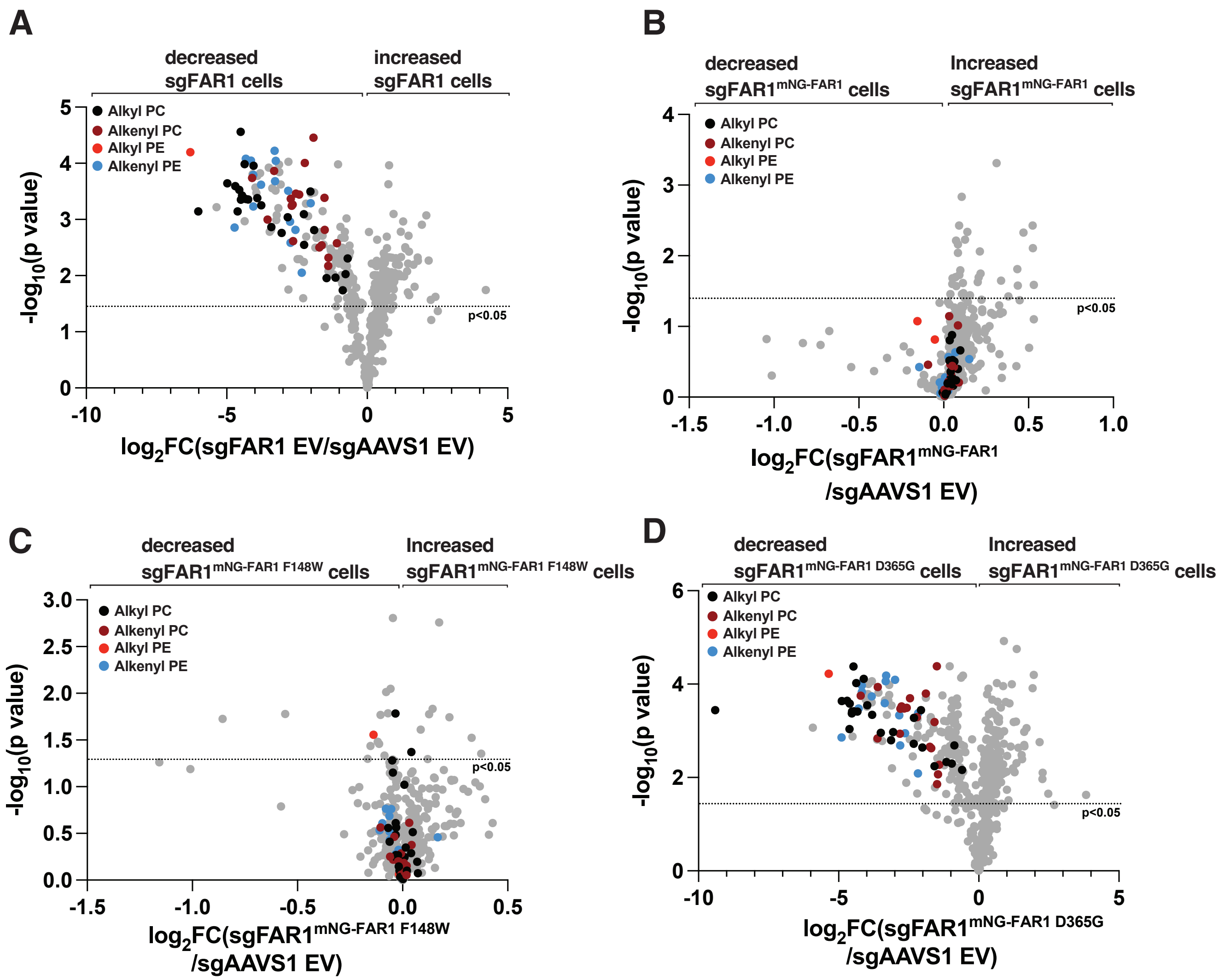

bioRxiv preprint doi: https://doi.org/10.64898/2026.09.28.755133; this version posted September 29, 2026. The copyright holder for this preprint (which was not certified by peer review) is the author/funder, who has granted bioRxiv a license to display the preprint in perpetuity. It is made available under a CC-BY 4.0 International license.

Supplementary Figure 13

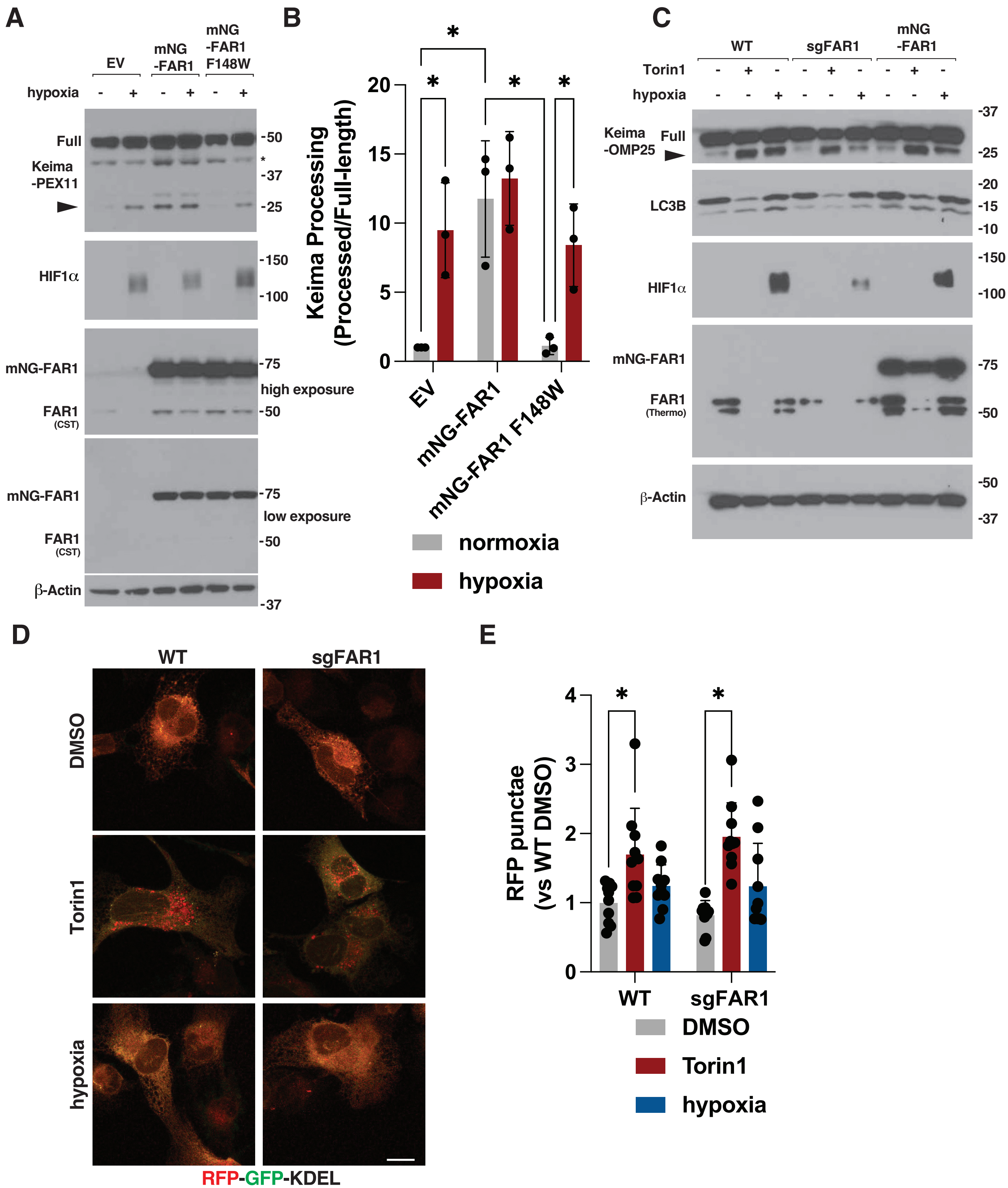

bioRxiv preprint doi: https://doi.org/10.64898/2026.09.28.755133; this version posted September 29, 2026. The copyright holder for this preprint (which was not certified by peer review) is the author/funder, who has granted bioRxiv a license to display the preprint in perpetuity. It is made available under a CC-BY 4.0 International license.

Supplementary Figure 14

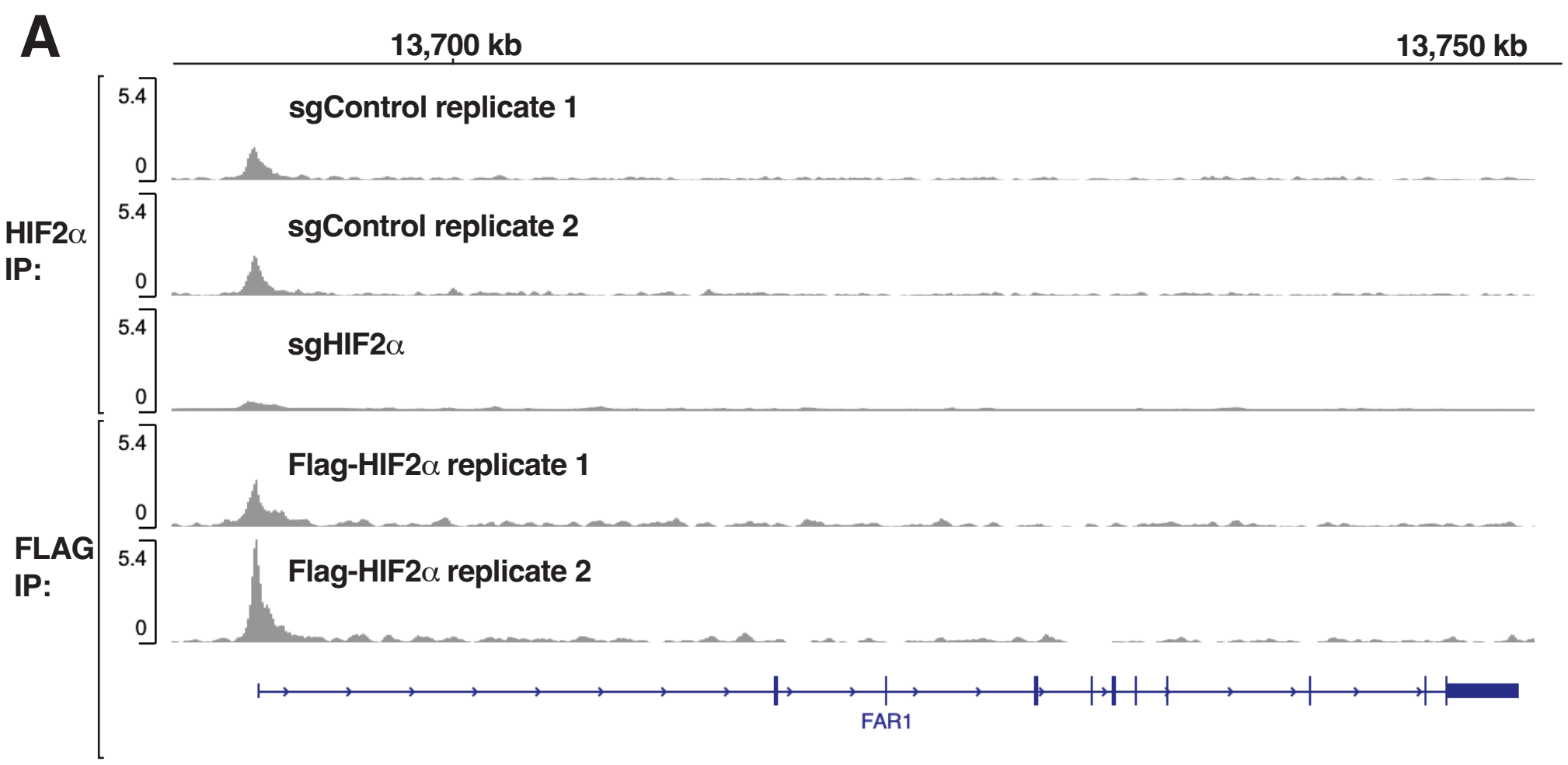

bioRxiv preprint doi: https://doi.org/10.64898/2026.09.28.755133; this version posted September 29, 2026. The copyright holder for this preprint (which was not certified by peer review) is the author/funder, who has granted bioRxiv a license to display the preprint in perpetuity. It is made available under a CC-BY 4.0 International license.

Supplementary Figure 15

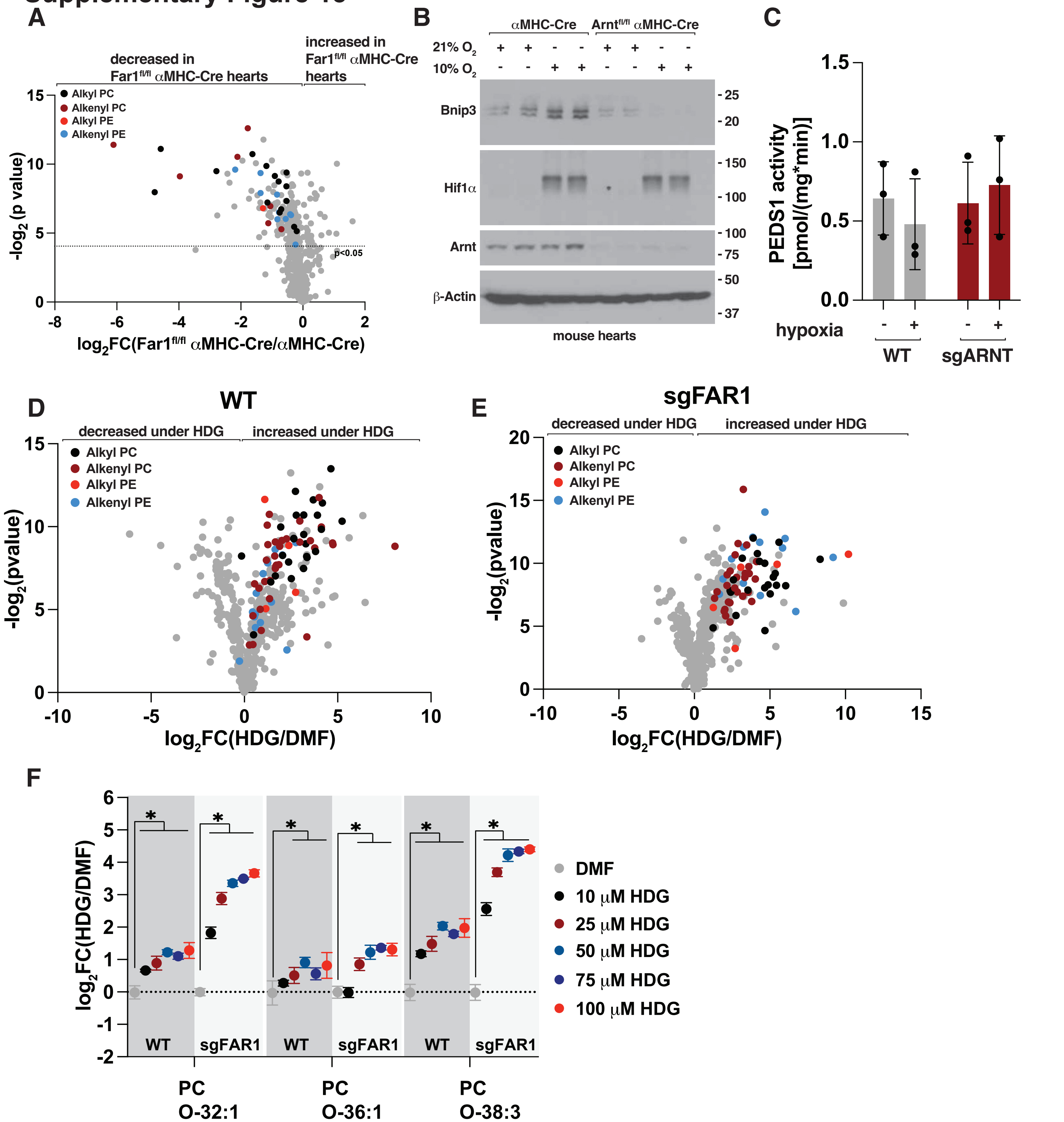

bioRxiv preprint doi: https://doi.org/10.64898/2026.09.28.755133; this version posted September 29, 2026. The copyright holder for this preprint (which was not certified by peer review) is the author/funder, who has granted bioRxiv a license to display the preprint in perpetuity. It is made available under a CC-BY 4.0 International license.

Supplementary Figure 16

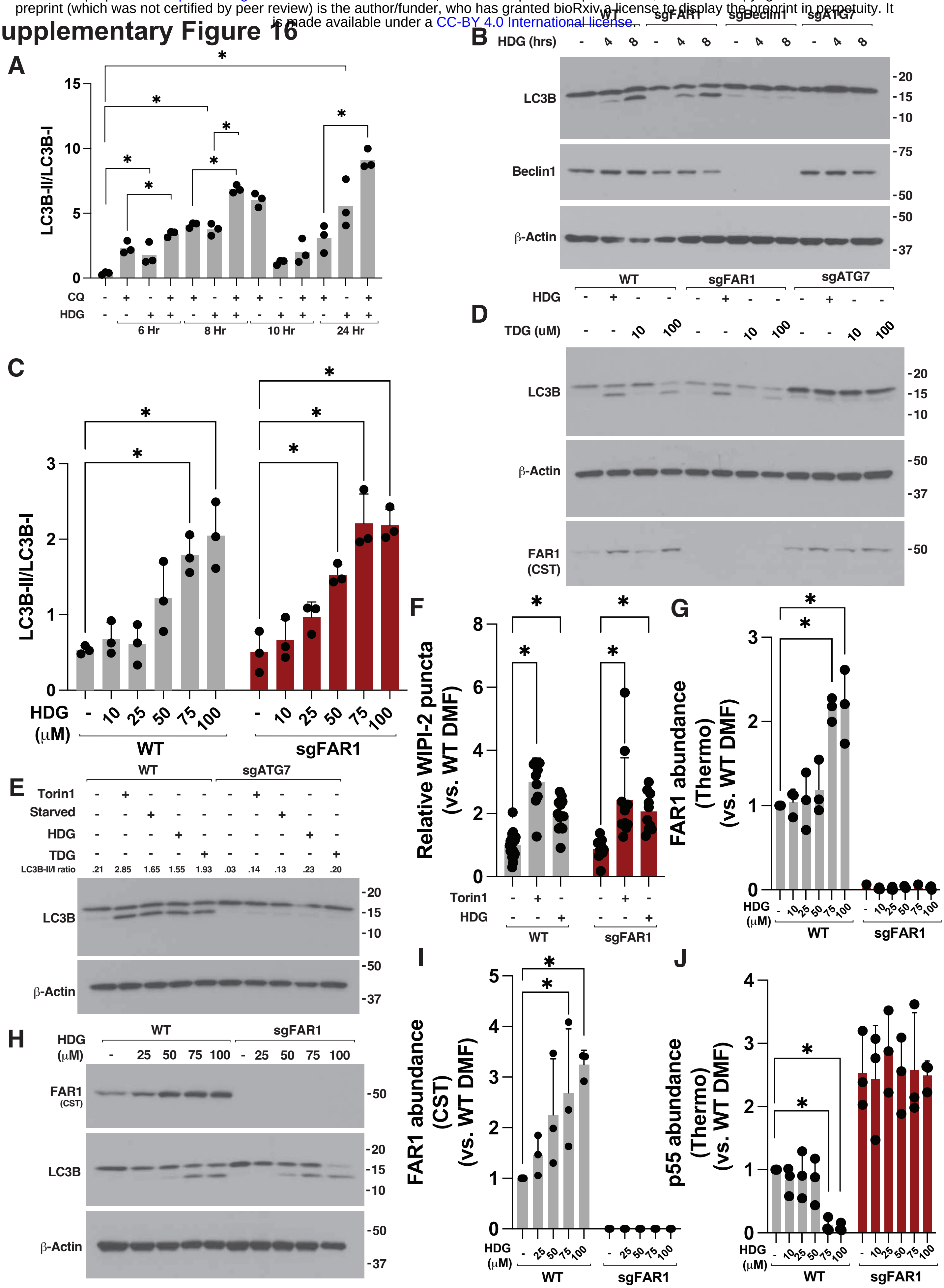

bioRxiv preprint doi: https://doi.org/10.64898/2026.09.28.755133; this version posted September 29, 2026. The copyright holder for this preprint (which was not certified by peer review) is the author/funder, who has granted bioRxiv a license to display the preprint in perpetuity. It is made available under a CC-BY 4.0 International license.

Supplementary Figure Legends

Supplementary Figure 1. Validation of Peroxisome-Keima reporter system. (A) Schematic of Keima Peroxisome reporter system that allows for monitoring of lysosomal delivery of autophagy cargo. The acidic environment of the lysosome causes an increase in the ratio of 561 nm/488 nm excitation. Once the Keima-peroxisome fusion protein enters the lysosome, the Keima-fusion is processed to degrade the fusion protein, while the Keima (and any peptidic remnants left from the fusion partner) is resistant to the lysosomal proteases and maintains its fluorescence in the lysosome. **(B)** Immunofluorescence analysis of human AC16 cardiomyocytes stably expressing Keima-FLAG-PEX11. Scale bar indicates 10 um, inset scale bar indicates 1 um. **(C)** Immunoblot analysis of human U2OS osteosarcoma cells stably expressing Keima-PEX11. Where indicated, cells were grown at 0.1% O2 (hypoxia), treated with 250 nM Torin1, 1 uM SAR405 or normoxia and treated with DMSO for 18 hours. Full indicates full-length Keima-fusion protein and arrow indicates liberated Keima protein. **(D)** Immunoblot analysis of human U2OS osteosarcoma cells stably expressing Keima-PEX26. Where indicated, cells were grown at 0.1% O2 (hypoxia), treated with 100 uM FG-4497, 1 uM TAK-243 (E1 inhibitor), 1 uM MG132, 1 uM SAR405, or normoxia and treated with DMSO for 18 hours. Full indicates full-length Keima-fusion protein and arrow indicates liberated Keima protein.

Supplementary Figure 2. ATG7 is necessary for hypoxia-induced pexophagy. (A-C) Immunoblot analysis of wild-type (WT), sgATG7 (A-B), sgVHL (B), and sgARNT (C) human AC16 cardiomyocytes. In (A), cells are stably expressing Keima-PEX11 and grown at 0.1% O2 (hypoxia) or normoxia for 18 hours. *indicates non-specific band. In (B), cells were grown at 0.5% O2 (hypoxia) or normoxia for indicated time points (hours) prior to cell lysis. In (C), cells were grown at 0.1% O2 (hypoxia) or normoxia for 36 hours prior to cell lysis. **(D)** Immunoblot analysis of $Atg7^{+/+}$-αMHC-Cre and $Atg7^{fl/fl}$-αMHC-Cre mouse hearts.

Supplementary Figure 3. Loss of NBR1, BNIP3, or FUNDC1 does not block hypoxia -induced pexophagy in human AC16 cardiomyocytes. (A-B) Immunoblot analysis of wild-type (WT) and sgNBR1 human AC16 cardiomyocytes. In (A), cells were treated with 500 nM Bafilomycin A1 (BafA1), grown at 0.1% O2 (hypoxia), or normoxia and treated with DMSO for 18 hours. In (B), cells were stably expressing Keima-PEX11 or empty vector (EV). Where indicated, cells were treated with 250 nM Torin1, grown at 0.1% O2 (hypoxia), or normoxia and treated with DMSO for 18 hours. Full indicates full-length Keima-fusion protein and arrow indicates liberated Keima protein. **(C)** Immunoblot analysis of wild-type (WT), sgATG7, sgBNIP3, and sgFUNDC1 human AC16 cardiomyocytes. Where indicated, cells were grown at 0.1% O2 (hypoxia) or normoxia for 36 hours prior to cell lysis.

Supplementary Figure 4. Identification of FAR1 via quantitative proteomics. (A) Immunoblot analysis of wild-type and sgATG7 human U2OS osteosarcoma cells. Where indicated, cells were grown at 0.1% O_2 (hypoxia), treated with Torin1 (250 nM), or treated with DMSO and grown at normoxia for 18 hours prior to cell lysis. **(B)** U2OS proteome analysis of total peptides and total proteins identified. **(C)** Principal component analysis

bioRxiv preprint doi: https://doi.org/10.64898/2026.09.28.755133; this version posted September 29, 2026. The copyright holder for this preprint (which was not certified by peer review) is the author/funder, who has granted bioRxiv a license to display the preprint in perpetuity. It is made available under a CC-BY 4.0 International license.

of tandem mass tagging (TMT) analysis of wild-type and sgATG7 U2OS cells grown under 0.1% O_2 (hypoxia), treated with Torin1 (250 nM) or treated with DMSO and grown at normoxia for 18 hours prior to cell lysis. **(D)** Immunoblot analysis of $Atg7^{+/+}$-αMHC-Cre and $Atg7^{fl/fl}$-αMHC-Cre mouse hearts. Far1 immunoblot indicates which Far1 antibody was used to detect Far1. **(E)** Immunoblot analysis of $Atg7^{+/+}$-Acta-Cre and $Atg7^{fl/fl}$ Acta-Cre mouse quadricep muscles. **(F)** Densitometry analysis of Far1 abundance shown in (E). N=6. *indicates P<0.05. **(G)** Far1 mRNA analysis in mouse hearts isolated from $Atg7^{+/+}$-αMHC-Cre and $Atg7^{fl/fl}$-αMHC-Cre mice. N>3, NS=non-significant.

Supplementary Figure 5. FAR1 localization at the peroxisome. (A) Confocal microscopy analysis of mouse neonatal cardiomyocytes. Scale bar indicates 10 um. **(B)** Immunoblot analysis of subcellular fractionation in wild-type and sgFAR1 human AC16 cardiomyocytes. WCE, whole cell extract. Cytosolic, cytosolic fraction. Membrane, membrane fraction. Far1 immunoblot indicates which Far1 antibody was used to detect Far1. **(C)** Confocal microscopy analysis of human AC16 cardiomyocytes stably expressing mNeonGreen-Far1 (mNG-Far1) and labeled with 100 nM MitoTracker Deep Red. Scale bar indicates 10 um.

Supplementary Figure 6. FAR1 is degraded via the lysosome. (A) Immunoblot analysis of wild-type and sgFAR1 mouse neonatal cardiomyocytes. Where indicated, cells were treated with 250 nM Torin1, 30 uM Chloroquine (CQ), or DMSO for 18 hours prior to cell lysis. Arrow indicates FAR1. Far1 immunoblot indicates which Far1 antibody was used to detect Far1. **(B)** Immunoblot analysis of wild-type and sgFAR1 human 293FT cells. Where indicated, cells were deprived of nutrients (starvation), treated with 30 uM Chloroquine (CQ), or DMSO for 18 hours prior to cell lysis. Far1 immunoblot indicates which Far1 antibody was used to detect Far1. **(C)** Immunoblot analysis of mouse C2C12 myoblasts. Where indicated, cells were treated with 250 nM Torin1, 500 nM Bafilomycin A1 (BafA1), 30 uM Chloroquine (CQ), or DMSO for 18 hours prior to cell lysis. Arrow indicates Far1. Far1 immunoblot indicates which Far1 antibody was used to detect Far1. **(D)** Immunoblot analysis of wild-type and sgFAR1 human 293FT cells. Where indicated, cells were treated with 250 nM Torin1, 500 nM Bafilomycin A1 (BafA1), 1 uM MG132, or DMSO for 18 hours prior to cell lysis. Far1 immunoblot indicates which Far1 antibody was used to detect Far1. **(E)** *FAR1* mRNA analysis in human AC16 cardiomyocytes. Where indicated, cells were treated with 30 uM Chloroquine (CQ), 1 uM MG132, or DMSO for 18 hours prior to RNA extraction. N=3, NS=non-significant. **(F)** Confocal microscopy analysis of sgFAR1 293FT cells stably expressing mNG-FAR1. Scale bar indicates 10 um, inset scale bar indicates 1 um. **(G)** Image analysis of sgFAR1 human 293FT cells stably expressing mNG-FAR1 and labeled with lysotracker (LT) shown in Figure 2F. Where indicated, cells were grown at 0.1% O_2 (hypoxia) or normoxia prior to imaging. N=10, *P<0.05. **(H)** Image analysis of sgFAR1 human AC16 cells stably expressing mNG-FAR1 and labeled with lysotracker (LT) shown in Figure 2G. Where indicated, cells were starved of nutrients, treated with 30 uM Chloroquine (CQ), or DMSO for 18 hours prior to imaging. N=10, *P<0.05.

bioRxiv preprint doi: https://doi.org/10.64898/2026.09.28.755133; this version posted September 29, 2026. The copyright holder for this preprint (which was not certified by peer review) is the author/funder, who has granted bioRxiv a license to display the preprint in perpetuity. It is made available under a CC-BY 4.0 International license.

Supplementary Figure 7. FAR1 binds LC3B. (A) Immunoblot analysis of anti-FLAG immunoprecipitates isolated from cardiomyocytes stably expressing FLAG-LC3B K51A or FLAG-LC3B. Where indicated, cells were treated with 30 uM Chloroquine (CQ), grown at 0.1% O_2 (hypoxia), or treated with DMSO and grown at normoxia for 18 hours prior to cell lysis. Arrow indicates Far1. WCE, whole cell extract. Far1 immunoblot indicates which Far1 antibody was used to detect Far1. **(B)** Immunoblot analysis of anti-FLAG immunoprecipitates isolated from AC16 cells stably expressing FLAG-LC3B and mNG-FAR1, mNG-FAR1 D365G, or empty vector (EV). WCE, whole cell extract.

Supplementary Figure 8. FAR1 F148W localizes to the peroxisome. (A) Confocal microscopy analysis of sgFAR1 human AC16 cardiomyocytes stably expressing mNeonGreen-Far1 F148W (mNG-Far1 F148W) and mRFP-PEX26. Scale bar indicates 10 um.

Supplementary Figure 9. FAR1 is necessary for hypoxia induced peroxisome degradation. (A) Total-, C16-, and C18-plasmalogens (nmol/mg protein) in wild-type and sgFAR1 human AC16 cardiomyocytes. N=3, *indicates P<0.05. **(B)** Volcano plots comparing lipid intensities in wild-type and sgFAR1 AC16 cardiomyocytes. Black dots indicate alkyl (ether) phosphatidylcholine (PC), dark red dots indicate alkenyl PC (plasmalogens), red dots indicate alkyl (ether) phosphotidylethanolamine (PE), light blue dots indicate alkenyl PE (plasmalogens) species. Dotted line indicates P<0.05. **(C)** Densitometry analysis of Keima-processing assay as shown in wild-type and sgFAR1 human AC16 cardiomyocytes stably expressing Keima-PEX11. N=3, *indicates P<0.05. **(D)** Immunoblot analysis of wild-type and sgFAR1 human AC16 cardiomyocytes stably expressing Keima-PEX26. Where indicated, cells were grown at 0.1% O2 (hypoxia) or normoxia for 18 hours prior to cell lysis. Full indicates full-length Keima-fusion protein and arrow indicates liberated Keima protein. **(E-F)** Confocal microscopy (E) and image analysis (F) of wild-type (WT) and sgFAR1 human AC16 cardiomyocytes. Where indicated, cells were grown at 0.1% O2 (hypoxia) or normoxia for 18 hours prior to cell fixation. N=10, *P<0.05. Scale bar indicates 10 um, inset scale bar indicates 1 um. **(G)** Immunoblot analysis of wild-type (WT) and sgFAR1 human AC16 cardiomyocytes. Where indicated, cells were treated with 250 nM Torin1, 30 uM Chloroquine (CQ), or DMSO for 18 hours prior to cell lysis. Far1 immunoblot indicates which Far1 antibody was used to detect Far1. **(H)** Immunoblot analysis of wild-type (WT) and sgFAR1 human AC16 cardiomyocytes. Where indicated, cells were treated with 500 uM Dimethyloxalylglycine (DMOG), grown at 0.1% O2 (hypoxia), treated with 100 uM FG-4592, or normoxia and treated with DMSO for 8 hours prior to cell lysis. Arrow indicates FAR1 while * indicates non-specific band. Far1 immunoblot indicates which Far1 antibody was used to detect Far1.

Supplementary Figure 10. FAR1 and its role in PEX1-mediated pexophagy. (A-B) Confocal microscopy (A) and image analysis (B) of PEX14-positive peroxisomes in wild-type and sgFAR1 human AC16 cells stably expressing sgRNAs targeting sgAAVS1 (control) or sgPEX1. N>10, * indicates P<0.05.

Supplementary Figure 11. Far1 co-dependency mapping. (A) FAR1 co-dependency mapping using DepMap. **(B)** *AGPS*, *FAR2*, *GNPAT*, and *PEDS1* mRNA analysis, as

bioRxiv preprint doi: https://doi.org/10.64898/2026.09.28.755133; this version posted September 29, 2026. The copyright holder for this preprint (which was not certified by peer review) is the author/funder, who has granted bioRxiv a license to display the preprint in perpetuity. It is made available under a CC-BY 4.0 International license.

determined by real-time polymerase chain reaction, in wild-type and sgFAR1 human AC16 cardiomyocytes. N=3, *P<0.05. **(C)** Immunoblot analysis of wild-type and sgFAR1 human AC16 cardiomyocytes. Arrow indicates FAR1 while * indicates non-specific band. Far1 immunoblot indicates which Far1 antibody was used to detect Far1. **(D)** PEX7 co-dependency mapping using DepMap. **(E-F)** Densitometry analysis of Keima-processing assay as shown in wild-type, sgAGPS (E) and sgPEX7 (F) human AC16 cardiomyocytes stably expressing Keima-PEX11. N=3, *indicates P<0.05. **(G)** Immunoblot analysis of wild-type and sgACSL4 human AC16 cells stably expressing Keima-PEX11. Full indicates full-length Keima-fusion protein and arrow indicates liberated Keima protein. **(H-I)** Immunoblot analysis of wild-type (WT), sgPEX7, sgAGPS (H), and sgACSL4 (I). Where indicated, cells were treated with 100 uM FG-4592, grown at 0.1% O2 (hypoxia), or treated with DMSO and grown at normoxia for 8 hours prior to cell lysis.

Supplementary Figure 12. Lipidomic analysis sgFAR1 cells re-expressing FAR1 F148W or FAR1 D365G. (A-D) Volcano plots comparing lipid intensities in wild-type sgAAVS1 cells stably expressing empty vector (EV) and sgFAR1 293FT (A) cells stably expressing EV (A), mNG-FAR1 (B), mNG-FAR1 F148W (C), mNG-FAR1 D365G (D). All volcano plots are compared against sgAAVS1 293FT cells expressing EV. Black dots indicate alkyl (ether) phosphatidylcholine (PC), dark red dots indicate alkenyl PC (plasmalogens), red dots indicate alkyl (ether) phosphotidylethanolamine (PE), light blue dots indicate alkenyl PE (plasmalogens) species. Dotted line indicates P<0.05.

Supplementary Figure 13. Analysis of hypoxia-induced pexophagy, mitophagy, and ER-phagy. (A) Immunoblot analysis of wild-type AC16 cardiomyocytes stably expressing Keima-PEX11 and mNG-FAR1, mNG-FAR1 F148W, or empty vector (EV). Where indicated, cells were grown under 0.1% O2 (hypoxia) or normoxia for 18 hours prior to cell lysis. Full indicates full-length Keima-fusion protein and arrow indicates liberated Keima protein. Far1 immunoblots indicate which Far1 antibody was used to detect Far1. **(B)** Densitometry analysis of Keima-processing assay as shown in wild-type human AC16 cardiomyocytes stably expressing Keima-PEX11 and mNG-FAR1, mNG-FAR1 F148W, or empty vector (EV). **(C)** Immunoblot analysis of wild-type (WT) and sgFAR1 human AC16 cardiomyocytes stably expressing Keima-OMP25. Where indicated, wild-type cardiomyocytes were stably expressing empty vector or mNeonGreen-Far1 (mNG-FAR1). Cells were treated with 250 nM Torin1, grown at 0.1% O2 (hypoxia), or treated with DMSO and grown at normoxia for 18 hours prior to cell lysis. Far1 immunoblot indicates which Far1 antibody was used to detect Far1. **(D-E)** Confocal microscopy (D) and RFP imaging analysis (E) of wild-type (WT) and sgFAR1 human AC16 cardiomyocytes stably expressing RFP-GFP-KDEL. Where indicated, cells were treated with 250 nM Torin1, grown at 0.1% O_2 (hypoxia), or grown at normoxia and treated with DMSO for 18 hours prior to imaging. N=10. *indicates p<0.05. Scale bar indicates 10 um.

Supplementary Figure 14. FAR1 is a direct HIF target. (A) FAR1 ChIP-Seq analysis of publicly available GSE253325 data. Representative Anti-HIF2α ChIP-seq tracks in 786-O cells that underwent CRISPR-based gene editing with a HIF2α sgRNA or a control sgRNA (sgCtrl) and representative Anti-FLAG ChIP-seq tracks in 786-O cells in which a

bioRxiv preprint doi: https://doi.org/10.64898/2026.09.28.755133; this version posted September 29, 2026. The copyright holder for this preprint (which was not certified by peer review) is the author/funder, who has granted bioRxiv a license to display the preprint in perpetuity. It is made available under a CC-BY 4.0 International license.

FLAG-HA epitope tag was inserted at the 5' end of the endogenous *EPAS1* open reading frame by CRISPR-HDR (FLAG-HIF2α).

Supplementary Figure 15. Ether lipids abundance under hypoxia and HDG. (A) Volcano plots comparing lipid intensities in αMHC-Cre and Far1$^{fl/fl}$ αMHC-Cre mouse hearts. **(B)** Immunoblot analysis of Arnt$^{+/+}$-αMHC-Cre and Arnt$^{fl/fl}$-αMHC-Cre mouse hearts. Where indicated, mice were housed at 10% O_2 (hypoxia) or 21% O_2 (atmospheric oxygen) for 7 days prior to euthanasia. **(C)** PEDS1 activity [pmol/(mg*min)] in wild-type and sgARNT human AC16 cardiomyocytes. Where indicated, cells were grown at 0.1% O_2 (hypoxia), or normoxia for 18 hours prior to cell harvesting. N=3. **(D-E)** Volcano plots comparing lipid intensities in wild-type (D) and sgFAR1 (E) human AC16 cardiomyocytes treated with 1-O-hexadecyl-*sn*-glycerol (100 uM, HDG) or DMF for 24 hours prior to lipid extraction. Black dots indicate alkyl (ether) phosphatidylcholine (PC), dark red dots indicate alkenyl PC (plasmalogens) species, red dots indicate alkyl (ether) phosphotidylethanolamine (PE), and light blue dots indicate alkenyl PE (plasmalogens) species. **(F)** Lipidomic profiling of alkyl PC species in wild-type and sgFAR1 human AC16 cardiomyocytes. Where indicated, cells were treated with 1-O-hexadecyl-sn-glycerol (HDG) at the indicated dose (uM) or DMF for 8 hours prior to lipid extraction. N=3, *P<0.05.

Supplementary Figure 16. Ether lipid precursors activate autophagy. (A) Densitometry analysis of LC3B-II/LC3B-I ratio from immunoblot assays. Where indicated, cell lysates were prepared from wild-type human AC16 cells treated with 100 uM 1-O-hexadecyl-*sn*-glycerol (HDG), 30 uM Chloroquine (CQ), or DMF for indicated time (hours, hrs). N=3, *indicates P<0.05. **(B)** Immunoblot analysis of wild-type (WT), sgFAR1, sgBeclin1, and sgATG7 human AC16 cardiomyocytes. Where indicated, cells were treated with 100 uM 1-O-hexadecyl-*sn*-glycerol (HDG) or DMF for indicated time (hours, hrs) prior to cell lysis. **(C)** Densitometry analysis of LC3B-II/LC3B-I ratio from immunoblot assays. Where indicated, cell lysates were prepared from wild-type human or sgFAR1 AC16 cells treated with indicated concentration of 1-O-hexadecyl-*sn*-glycerol (HDG) or DMF. N=3, *indicates P<0.05. **(D)** Immunoblot analysis of wild-type (WT), sgFAR1, and sgATG7 human AC16 cardiomyocytes. Where indicated, cells were treated with 100 uM 1-O-hexadecyl-*sn*-glycerol (HDG), indicated dose of 1-O-tetradecyl glycerol (TDG), or DMF for 8 hours prior to cell lysis. Far1 immunoblot indicates which Far1 antibody was used to detect Far1. **(E)** Immunoblot analysis of wild-type (WT) and sgATG7 human AC16 cardiomyocytes. Where indicated, cells were treated with 250 nM Torin1, starved of nutrients, treated with 100 uM 1-O-hexadecyl-*sn*-glycerol (HDG), treated with 100 uM 1-O-tetradecyl glycerol (TDG), or DMF for 8 hours prior to cell lysis. **(F)** Confocal microscopy and image analysis of WIPI-2 puncta in wild-type and sgFAR1 human AC16 cells. Where indicated, cells were treated with 250 nM Torin1, 100 uM 1-O-hexadecyl-*sn*-glycerol (HDG), or DMF for 8 hours prior to cell fixation. N>10, *indicates P<0.05. **(G)** Densitometry analysis of FAR1 abundance using the Thermo PA5-53585 antibody from immunoblot assays. Where indicated, cell lysates were prepared from wild-type and sgFAR1 human AC16 cells treated with indicated dose of 1-O-hexadecyl-*sn*-glycerol (HDG) or DMF for

bioRxiv preprint doi: https://doi.org/10.64898/2026.09.28.755133; this version posted September 29, 2026. The copyright holder for this preprint (which was not certified by peer review) is the author/funder, who has granted bioRxiv a license to display the preprint in perpetuity. It is made available under a CC-BY 4.0 International license.

24 hours. N=3, *indicates P<0.05. **(H)** Immunoblot analysis of wild-type (WT) and sgFAR1 human AC16 cardiomyocytes. Where indicated, cells were treated with indicated dose of 1-O-hexadecyl-*sn*-glycerol (HDG) or DMF for 24 hours. FAR1 immunoblot indicates which FAR1 antibody was used to detect FAR1. **(I)** Densitometry analysis of FAR1 abundance using the CST FAR1 antibody from immunoblot assays. Where indicated, cell lysates were prepared from wild-type and sgFAR1 human AC16 cells treated with indicated dose of 1-O-hexadecyl-*sn*-glycerol (HDG) or DMF for 24 hours. N=3, *indicates P<0.05. **(J)** Densitometry analysis of 55 kDa band (p55) abundance detected using FAR1 Thermo PA5-53585 antibody from immunoblot assays. Where indicated, cell lysates were prepared from wild-type and sgFAR1 human AC16 cells treated with indicated dose of 1-O-hexadecyl-*sn*-glycerol (HDG) or DMF for 24 hours. N=3, *indicates P<0.05.
